# Highly Sensitive and Practical Detection of Plant Viruses via Electrical Impedance of Droplets on Textured Silicon-Based Devices

**DOI:** 10.3390/s16111946

**Published:** 2016-11-18

**Authors:** Marianna Ambrico, Paolo Francesco Ambrico, Angelantonio Minafra, Angelo De Stradis, Danilo Vona, Stefania R. Cicco, Fabio Palumbo, Pietro Favia, Teresa Ligonzo

**Affiliations:** 1 CNR NANOTEC—Istituto di Nanotecnologia, Via Amendola 122, Bari 70126, Italy; paolofrancesco.ambrico@cnr.it (P.F.A.); fabio.palumbo@cnr.it (F.P.); 2 CNR IPSP—Istituto per la Protezione Sostenibile delle Piante, UoS Bari, Via Amendola 165, Bari 70126, Italy; angelantonio.minafra@ipsp.cnr.it (A.M.); angelo.destradis@ipsp.cnr.it (A.D.S.); 3 Dipartimento di Chimica, Università degli Studi di Bari, Via Orabona 4, Bari 70125, Italy; danilo.vona@gmail.com (D.V.); pietro.favia@uniba.it (P.F.); 4 CNR ICCOM—Istituto di Chimica dei Composti OrganoMetallici, Via Orabona 4, Bari 70125, Italy; cicco@ba.iccom.cnr.it; 5 Dipartimento Interateneo di Fisica, Università degli Studi di Bari, Via Orabona 4, Bari 70125, Italy; ligonzo@fisica.uniba.it

**Keywords:** plant viruses, EIS, label-free detection, ToMV, TYMV, droplet-based device, surface texturing

## Abstract

Early diagnosis of plant virus infections before the disease symptoms appearance may represent a significant benefit in limiting disease spread by a prompt application of appropriate containment steps. We propose a label-free procedure applied on a device structure where the electrical signal transduction is evaluated via impedance spectroscopy techniques. The device consists of a droplet suspension embedding two representative purified plant viruses i.e., *Tomato mosaic virus* and *Turnip yellow mosaic virus*, put in contact with a highly hydrophobic plasma textured silicon surface. Results show a high sensitivity of the system towards the virus particles with an interestingly low detection limit, from tens to hundreds of attomolar corresponding to pg/mL of sap, which refers, in the infection time-scale, to a concentration of virus particles in still-symptomless plants. Such a threshold limit, together with an envisaged engineering of an easily manageable device, compared to more sophisticated apparatuses, may contribute in simplifying the in-field plant virus diagnostics.

## 1. Introduction

The development of modern multifunctional biologically-oriented platforms for a sensitive, reliable, and rapid detection of plant viruses plays a critical role in virus disease management. Although a single diagnostic test may provide adequate information on the identity of a virus, a combination of methods is generally needed for unequivocal diagnosis. Thus, methods for detection of plant viruses should be sensitive, specific, and should be completed within a relatively short period of time at a low cost.

Generally, diagnostic techniques in virus detection fall into two categories due to the intrinsic properties of the virus itself. Methods are based on the detection of coat proteins and genomic nucleic acids as enzyme-linked immunosorbent assays (ELISA) and immunoblotting (DIBA), whose virus protein limit of detection (LOD) is from 1 to 10 ng/mL of sap, and on the reverse transcription polymerase chain reaction (RT-PCR), the latter being more sensitive than other methods reaching a LOD of 1 fg/mL of virus RNA [1,2,3,4]. Nowadays, polymerase chain reaction (PCR) is a particularly attractive technique for the diagnosis of plant viruses because it is able to amplify the target nucleic acid starting from an extremely low concentration in a complex mixture of heterologous sequences [5,6].

In recent years, biosensor-based techniques were developed for the detection of intact plant viruses. Quartz crystal microbalance (QCM) immunosensor demonstrated the potential to detect as low as 1.0 ng each of two orchid viruses [7]. Surface plasmon resonance (SPR)-based biosensors were recognized as an efficient technique for the rapid diagnosis through complexing with specific antibodies of *Tobacco mosaic virus* (TMV) [8], and *Asian rust virus* on the soybean leaf with a declared LOD of μg/mL [9].

Nevertheless, all the sensors developed until now are quite expensive, highly complex, require many steps, as well as expert operators and furthermore, and are not suitable for rapid in-field testing.

Nowadays, the impedance bio-sensing technique represents a valuable alternative as it can shed light on the changes in the electrochemistry during the analyte detection. In spite of all of the other techniques, the impedance-based methods have the advantages to be, in most cases, label-free and easily miniaturized with a simple design at low cost. Recently, label-free identification of human and animals virus-based suspension via capacitance based measurements and using electrical parameters was recently performed [10,11,12]. Such techniques have also been introduced in plant virus detection. As an example, the development of contactless dielectric microsensors containing integrated molecular imprinted polymers (MIPs) as the sensing layer to detect viral contamination of TMV was reported [13]. However, microfluidic conditions, sensor geometries, and choice of polymeric material used to fabricate the MIP make this system not so suitable for the on-field virus detection. Recently, the detection of *Prunus necrotic ringspot virus* in plant extracts was obtained by electrical impedance spectroscopy (EIS)-based immunosensing technique via the virus antigen-antibody interaction, with the latter immobilized on a modified glassy carbon electrode [14]. In this case, the lowest attained detection limit was in the range of μg/mL of plant extracts. Although all of these techniques do not require expensive reagents, an optimal antibody orientation and density is recommended for a specific virus detection.

The innovative droplet-on-a-surface configuration recently opened new perspectives in the design of highly sensitive label-free lab-on-a-chip EIS based devices for virus detection. Droplets were displaced on microfluidic devices and acted as chemical and biological reactors where changes in droplet impedance allowed the analysis of processes occurring inside [15,16]. A high impedance hybrid droplet-solid interface-lipid bilayer membrane was developed through controlling the contact area between an aqueous droplet and an electrode. The resulting artificial cell membrane system was suitable for ion channel study and drug screening [17]. Furthermore, numerical/analytical theory have been developed for studying the impedance evolution of droplets involving various analyte concentrations when deposited on a surface defined by coplanar electrodes [18]. An attomolar (aM) scale limit was achieved in DNA hybridization by using microliter droplet in distilled water on a nanotextured superhydrophobic electrode array [19]. Within this context, this work is aiming to present a low-cost, label-free, and totally chemistry-free platform for plant virus detection based on impedance spectroscopy. The application of the EIS as an alternative methodology in virus recognition turned in an interesting highly sensitive detection of plant viruses in aqueous buffers via a hybrid system, made up of a droplet on the high hydrophobic semiconducting surface.

## 2. Materials and Methods

### 2.1. Virus Description and Morphological Characterization

Two well-known viruses, *Turnip yellow mosaic virus* (TYMV) [20] and *Tomato mosaic virus* (ToMV) [21] were chosen as the model systems, due to their high stability and very different structure.

**Figure 1:**
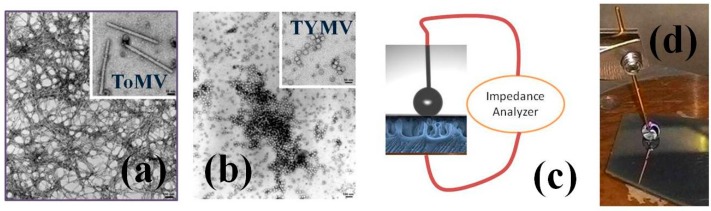
Transmission electron microscopy images of ToMV (**a**) and TYMV (**b**). Bar sizes are 500 nm and 10 nm in the main pictures and in the insets, respectively; (**c**) Simplified sketch of the droplet/ textured p-type silicon (T-pSi)-based device and the connection to the measurement setup; (**d**) Photograph representing the typical virus particles-in-solution droplet on top of the highly hydrophobic T-pSi surface and one of the two needle probes for the connection to the experimental setup (see text).

*Tomato mosaic virus* [21] has a rod-like structure due to an assembly of 2130 identical coat protein (CP) subunits of 18 kDa, around a single-stranded RNA. Each CP subunit consists of 158 amino acids with acetylated N-termini. The virus particle is ca. *L* = 300 nm long and *d* = 18 nm in diameter, with a 4 nm wide inner channel (Figure 1a).

*Turnip yellow mosaic virus* is an icosahedral virus having a *Ø* = 30 nm diameter and a higher proportion of nucleic acid. (Figure 1b) [17]. Its virion is made of 180 protein subunits of about 20 kDa organized in 32 morphological units, and the genomic RNA has a molecular weight of 2 × 10^6^ Da. Empty virion shells are naturally produced in the infected plant, giving evidence that strong protein-protein interactions are fundamental for virion stability (Figure 1b).

TYMV and ToMV have isoelectric points (pI) of 3.7 and 4.5, respectively [20,21]. Consequently, as a general rule, when buffered in solution with pH > pI (pH < pI) they show a negatively- (positively-) charged surface. The nano-size and different magnitude of virus geometrical parameters implies that they can be considered as nanoparticles with different aspect ratio, *L*/*d*.

### 2.2. Virus Extraction, Characterization, and Suspension Preparation

Leaves of young seedlings of *Nicotiana tabacum* and *Chinese cabbage* were mechanically inoculated by ToMV and TYMV, respectively, by gently rubbing the sap from infected tissues macerated in 100 mM tris(hydroxymethyl)aminomethane chlorohydrate (TRIS-HCl) buffer, pH = 8.0. Both viruses were purified as in Foster and Taylor [22].

Infected leaves at 15 days post inoculation (*dpi*) were crushed in a blender with ice-cold buffer, filtered and clarified with 0.4 vol chloroform/n-butanol (1:1). The mixture was stirred on ice for 15 min and centrifuged at 6000 rpm for 30 min. The supernatant was stratified on a 3 mL cushion of 30% sucrose in TRIS-HCl solution and centrifuged at 30,000 rpm for 2.5 h. The resulting pellets were re-suspended in the same buffer and subjected to a further similar round of centrifugation. The partially-purified viruses were finally re-suspended and stored indefinitely at 4 °C.

The morphological virus characterization was performed by transmission electron microscopy (TEM) observation by using a Morgagni 282D (Philips, Amsterdam, The Netherlands). The virus samples were prepared by depositing 20 μL of the resuspended particles on carbon-coated copper grids, stained for 4 min with 2% uranyl acetate water solution (Figure 1).

Both suspensions of partially-purified viruses were scanned for optical density reading (320 to 220 nm) on a Nanodrop 2000 (Thermo Fisher Scientific, Waltham, MA, USA). The different extinction coefficients of the viruses, which depend on their relative nucleic acid contents [23], were used to calculate the molar concentration (Table S1 in Supplementary Materials). Afterward, the virus concentration *ρ* underwent to several ten-fold dilutions starting from the stock suspension in a range from *ρ* = 1.0 mg/mL, corresponding to the molar concentration *ρ*_M_ of 25 nM for ToMV and 177 nM for TYMV, down to 1.0 pg/mL, corresponding to *ρ*_M_ of 25 aM in ToMV and 177 aM in TYMV.

### 2.3. Surface Texturing

P-doped crystalline silicon slabs (pSi) (15 × 15 mm^2^, polished wafers, with resistivity 1–50 Ω·cm, 750 μm thick, provided by MEMC (St. Peters, MO, USA) were plasma textured in a radiofrequency (RF, at 13.56 MHz, Dressler Cesar 1330 (Advanced Energy, Fort Collins, CO, USA))-powered parallel plate reactor [24,25]. The slices were placed on the RF bottom electrode at a temperature kept at 25 °C with a re-circulating chilling system. The texturing was obtained by optimized maskless plasma etching. Details on the etching procedure can be found elsewhere [24,25]. In particular, in this case tetrafluoroethylene was admitted at a flow rate of 20 sccm keeping the pressure at 80 mTorr and the plasma was ignited at 300 W for 20 min.

Before surface exposure of the droplet, a pre-treatment of textured p-type silicon (T-pSi) slabs was required in order to make these supports suitable as platforms for viral sample spotting. To this aim a fast washing with acetone and ethanol followed by rinsing with bi-distilled water and sonication for 1 min at maximum power kept micro-pollutants away. Finally, the clean silicon surfaces were put in a vacuum chamber for 6 h.

The surface water contact angle, WCA, was evaluated using the sessile drop method, from triplicate measurements onto different points of the sample, returning an average advancing WCA of 152° ± 6° and a receding one of 120° ± 4°, indicating a quite uniform hydrophobic surface.

### 2.4. Device Configuration and Measurement Procedure

The T-pSi slices were highly hydrophobic respect to the suspensions droplet on the overall region constituting the sensing area, i.e., the droplets displayed a spherical shape with a similar contact angle of the droplets (see Figure 1c). Similar tests on flat silicon or gold surfaces were unsuccessful since the untreated surfaces were only partially and in-homogenously hydrophobic. The inhomogeneous surface causes droplets to have varying or low contact angles onto the surfaces i.e., the geometrical capacitance would be varying depending on droplet positions onto the supports [18]. Therefore, these surfaces could not allow the determination of a set of impedance data suitable for an unambiguous extraction of a calibration curve, depending solely on the virus concentration [18].

The device constituted T-pSi (15 × 15 mm^2^) slices exposed to a set of droplets (7 μL each) of ToMV or TYMV in buffered TRIS-HCl in water suspension at pH 8.0 (Figure 1c,d). The droplets were gently placed on the surface by using high-precision graded micropipettes. Before droplet exposure, the T-pSi slices was glued on a copper plate with the ohmic back contact realized by painting InGa paste on the brushed pSi back surface. The attained structure was likely a (liquid)-gated metal-insulator-semiconductor-like (MIS) structure, whose ‘liquid insulating gate’ was represented by the droplet (see Figure 1c).

### 2.5. Impedance Measurements

The impedance spectra were recorded on the droplet-on-TpSi structure by using a NOVOCONTROL impedance analyzer. The AC voltage signal *V*_AC_ was fixed at 30 mV while the frequency range was between *f* = 0.1 Hz and *f* = 10 MHz. Due to the selected range, the spectra recording lasted around three minutes. The electrical connection of the virus based droplet on the T-pSi system to the impedance analyzer was through two probe holders mounting gold plated needles connected to the analyzer (Figure 1c,d).

To account for possible impedance changes due to statistical variation due to either suspension or of the T-pSi wetting features, the impedance spectra were read out by choosing three or five different positions onto the T-pSi surface, depending on silicon slices dimensions. At each position, the surface was exposed to droplets with increasing virus molar concentration, *ρ*_M_, starting from those including blank TRIS-HCl alone. After collecting each impedance spectra, the droplet under test was withdrawn and substituted, in the same position, with a new one containing a higher virus concentration. The new droplet was verified to keep the same aspect ratio after each measurement, meaning no changes in the droplet shape and that of the contact angle in the selected position on the T-pSi slice after each exposure to the droplet.

**Figure 2:**
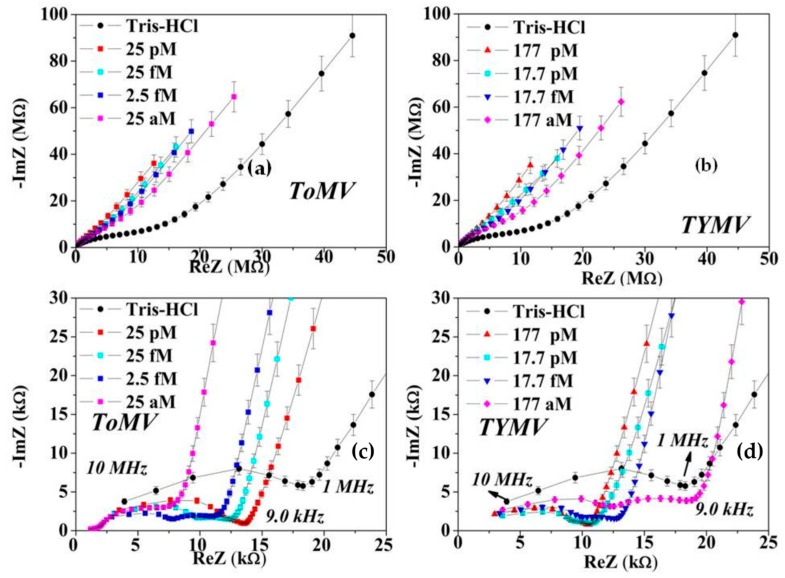
(**a**,**b**) Averaged NPs at *V*_DC_ = 0.0 V extracted from *Z* vs *f* spectra between 0.1 Hz and 10 MHz at four representative virus concentrations for ToMV and TYMV-in TRIS-HCl droplet suspension. (**c**,**d**) Detail of the data range in (**a**,**b**) in the higher frequency region. The frequency corresponding to the onset of the anomalous diffusion was 9.0 kHz in both virus-based suspensions and at all molar concentrations.

The *Z* vs. *f* spectra was calculated as the average of those collected at the same virus concentration on the elicited set of (three to five) droplets (see Figure 2). From the averaged *Z* vs. *f* spectra, the real, *ReZ*, and imaginary, *−ImZ*, part of impedance have been calculated and represented via the corresponding Nyquist plot (NPs, −*ImZ* vs. *ReZ*). The latter have been subsequently analyzed by modeling them with suitable equivalent circuits using a free downloadable EIS spectrum analyzer software [26,27].

The time evolution of NPs for a droplet residence time, *t*_r,_ onto the surface *t*_r_ = 20 min were collected both at *V*_DC_ = 0 and by adding a continuous negative bias (*V*_DC_ = −200 mV) to the AC voltage. The reasoning behind the choice of a residence time was due to the ascertained droplet volume and impedance stability after consecutively collecting the droplet impedance spectra for a similar residence time. The choice of collecting the impedance spectra under a negative DC voltage is aiming to induce a repulsive electric field drifting the negatively surface charged virus (pI (virus) < (pH(TRIS-HCl) = 8.0) from the gold electrode towards the textured silicon surface.

The equivalent circuits representing the NPs were designed by properly combining resistive (*R*), purely capacitive (*C*), constant-phase (*Z*_CPE_), and diffusive (*Z*_W_) circuital elements each representing a specific phenomenological contribution to the impedance [28].

## 3. Results

### 3.1. NPs Inspection

The homogenous surface wetting modification due to plasma texturing allowed the achievement of a highly hydrophobic textured silicon surface with a contact angle and preserved droplet aspect ratio even when changing the droplet positions on the surface.

The short duration of a single impedance spectrum (180 s) readout, and the very low evaporation rate of suspension buffer ruled out any significant droplet volume reduction and, consequently, any relevant changes in virus concentration. Rather, after 20 min and at *V*_DC_ = 0 V the NPs were still found unmodified in virus-including suspension droplets (see Figure S1 in Supplementary Materials). More specifically, the invariance of the first semi-circle, that relates to the droplet geometrical parameters (Figure S1), further support the elicited droplet volume stability.

The NPs at *V*_DC_ = 0 V of the virus-based suspension, displayed a significant variation respect to the blank one in the whole frequency range (Figure 2), a non-faradaic behavior and impedance controlled by diffusion mechanisms.

In the presence of viruses the diffusion turns to be more anomalous (Figure 2c,d), as evidenced by the steeper diffusion-related lines [29]. While the onset of diffusion was at around 1.0 MHz in the suspending TRIS-HCl medium, in the presence of viruses, it was shifted down to a value of 9.0 kHz, regardless the types and concentrations. At the lowest frequency (0.1 Hz), a concentration-related variation of the *ReZ* and −*ImZ* values was detected.

The NPs inspection in the range 10 MHz < *f* < 9.0 kHz, (Figure 2c,d), evidenced that, with respect to the buffer, a relevant left shift of the *ReZ* value at 9 kHz (the onset of the steep diffusion) was observed at the lowest concentration (corresponding to 1 pg/mL) in the case of ToMV, while in TYMV the steeper diffusion occurs at the same frequency but at higher *ReZ* values. Moreover, the higher the virus concentration, the more the ToMV (TYMV) curve shifts to the right (to the left).

**Figure 3:**
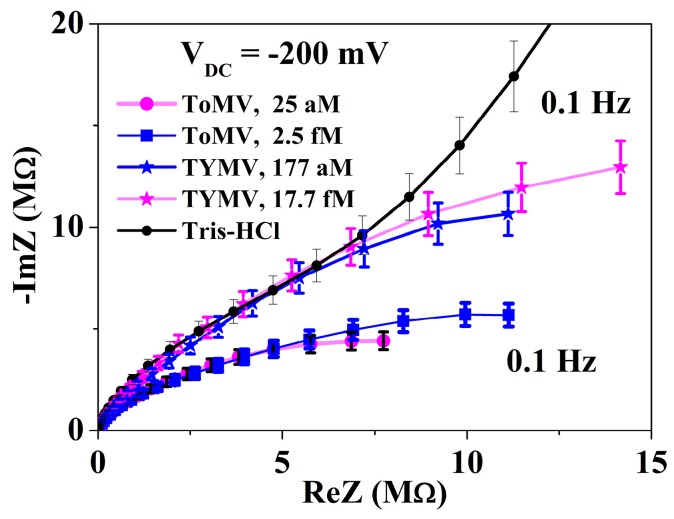
NPs read out at *V*_DC_ = −200 mV and *t* = 0 min, i.e., soon after the bias application in blank TRIS-HCl buffer and in ToMV and TYMV—Based suspension droplet at two representative concentrations (see legend). The NPs response was different depending on the virus embedded in the suspension and with respect to suspending medium.

Under the application of the continuous voltage, the NPs of virus suspensions displayed a more faradic behavior that was, furthermore, different on that of the biased blank one, the latter still retained a non-faradic trend (Figure 3). Moreover, the NPs were modifying both soon after the bias application and vs. time, still related to the virus type. The reasoning behind such results will be detailed later.

### 3.2. Calibration Curve Extraction and Virus Recognition via Real-Time Measurements

**Figure 4:**
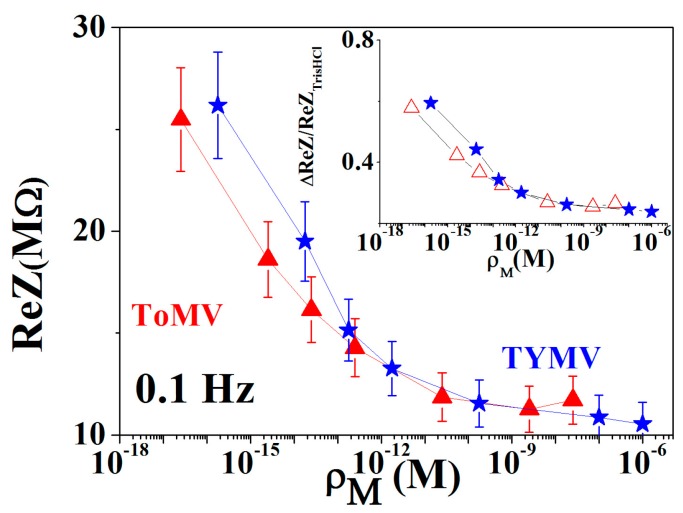
Calibration curves for virus quantification represented as *ReZ* at 0.1 Hz vs. the molar virus concentration in the droplet suspension. The inset represents the relative variation of the *ReZ* values respect to the blank TRIS-HCl.

The correlation between the NPs behavior with the molar concentration suggested to use them for the extraction of parameters suitable for virus quantification. By representing the *ReZ* values at 0.1 Hz vs. *ρ*_M_ (Figure 4) a linear dependence vs. the virus concentration in the range 100 aM < *ρ*_M_ < 10 fM was found, followed by a saturation behavior at higher concentrations. Therefore, the extracted calibration curves, one for each virus, were found particularly sensitive in quantifying very low virus concentrations starting from values where the plants are still symptomless. On the other hand, the saturation behavior at the highest concentration was attributed to the onset of the virus agglomeration and precipitation. Furthermore, it must be stressed that the concentration corresponding to the calibration curve saturation region (i.e., *ρ* ≥ μg/mL or in molar concentration equal to *ρ*_M_ > 0.1 pM and 1.0 pM in ToMV and TYMV, respectively), relates to infection stages where the plants are no longer symptomless with the disease already at an advanced stage.

The “water gated” MIS-like configuration confer to the droplet the role of the insulating part of the structure. Following the MIS device theory, the *ReZ* and −*ImZ* values are selective with respect to the charging and polarization of the insulator in the lower frequency ranges meaning, in this case charging and polarization of the droplet [28].

As a consequence, the returned lowering at 0.1 Hz of the *ReZ* and *ImZ* values with the concentration implies higher droplet ion charge-mediated conductivity, as could be expected by the increase of the virus charge density.

On the other hand, the behavior of the imaginary part −*ImZ* vs. the virus concentration, relates to the droplet/T-Si interface charging and polarization capabilities under the *AC* field [28] that can be estimated from rough data via the extraction of the capacitance, *C*. For a better evidence, the capacitance contribution *C* was extracted, in the overall measurement range, as a function of the frequency directly from NPs data following general calculation rules as [28]:

(1) $$Z=ReZ-jImZ=R-\frac{j}{2\pi fC}$$

(2) $$C=\frac{1}{(2\pi fsin\delta \sqrt{\left(ReZ^{2}+ImZ^{2}\right))}}$$

with *δ*, the phase angle, given by:

(3) $$\delta =\text{atan}\left(-\frac{ImZ}{ReZ}\right)$$

**Figure 5:**
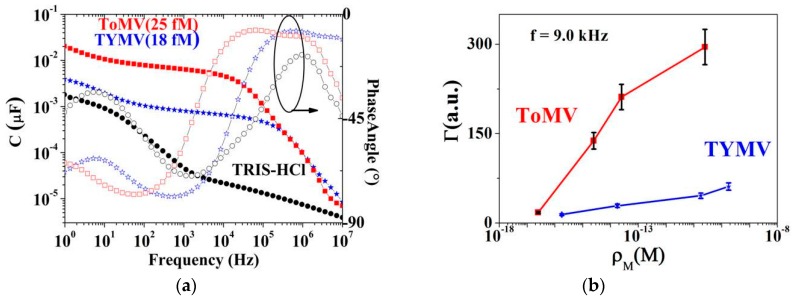
(**a**) Comparison between ToMV and TYMV capacitances (from Equation (2)) and phase angles frequency dispersion for a suspension at similar virus molarity; (**b**) *Γ* parameters extracted from capacitance values in Figure 5 at 9.0 kHz at the lowest virus molar concentrations corresponding to the plateau region in (**a**). The different capacitance variation vs virus types evidences the feasibility of virus identification by using this method. The full dataset has been reported in Figure S5 (Supplementary Materials).

Interestingly, the virus-based suspensions displayed a remarkable increase (up to three order of magnitude) of the capacitance at all frequencies (except at those >1 MHz) and a loss factor (i.e., of the phase angle) spectra different with respect to those of blank TRIS-HCl together with both a concentration and a virus-related behavior (Figure 5, Figure S2 in Supplementary Materials).

Since the measured capacitance *C* of the virus-based suspension, *C*_TRIS-HCl_, was higher than that of the buffer, we considered the elicited capacitance *C*_TRIS-HCl_ and that of the viruses, *C*_Vi_, acting as two parallel capacitors [12], likewise:

(4) $$C=C_{\text{TRIS}-\text{HCL}}+C_{\text{Vi}}$$

A proper frequency was selected where this effect was maximized, and the parameter *Γ* was defined as:

(5) $$\Gamma =\frac{C_{\text{Vi}}}{C_{\text{TRIS}-\text{HCl}}}=\frac{\left(C-C_{\text{TRIS}-\text{HCl}}\right)}{C_{\text{TRIS}-\text{HCl}}}$$

The *Γ* parameters were calculated by choosing the frequency of 9.0 kHz, i.e., lying in the plateau region of the *C* vs. *f* curves and maximizing the capacitance variation respect to that of the suspending medium. Interestingly, the substantially different behavior and values of the *Γ* parameters vs. the molar concentration on the virus type (Figure 5) suggested that ToMV and TYMV respond differently to the *AC* polarization field. These findings are particularly relevant since the highlight the definition of a possible marker (*Γ*) for the recognition of *AC*-responding viruses even at very low concentrations and, finally, directly extracted from rough experimental data [12].

The data in Figure 5a (and Figure S3) also elucidate better on the ToMV NPs stepped left shift of the *ReZ* at 9 kHz respect to the TYMV, at the onset of diffusion, and on the reversed shift when increasing the molar concentration.

The stepped shift in ToMV at the lowest concentration (1 pg/mL, i.e., 25 aM in ToMV and 177 aM in TYMV) can be explained not only as being due to a different electrical conductivity of the virus suspension, dictated by the number of the dielectric virus particles (i.e., seven times lower in ToMV with respect to TYMV) but also to a specific and peculiar virus behavior that will be better detailed in the next section.

In fact, both can be explained by examining the phase angle values at 9 kHz of each virus suspension at similarly low concentrations (Figure 5a and Figure S2). Considering Figure 5a, The ToMV phase angle is around −20° while the TYMV one is near −90°, meaning a lower resistive component in the former. These results descend from the ToMV aspect ratio (*L*/*d*~17(ToMV) > 1(TYMV)) and corresponding surface area higher than TYMV, both implying a higher electronic surface charge density available for conduction. 

Within this framework, it can be argument that the virus-depending behavior of the impedance (*ReZ* and *ImZ* at 9 kHz) vs. the concentration can also be a hint of a specific charge balance of the peculiar virus surface charges when interacting with the suspending aqueous medium, whose effect is strictly dependent on the intrinsic virus structure.

We recall that main and essential virus structure is made of a shell of folded protein sub-units that assemble with each other to maintain an outside hydrophilic surface, the latter particularly sensitive to any charge unbalance in the medium. The resulting surface electric charge is, therefore, very specific and dependent on the chemical composition in the primary amino-acid chain, and eventually remarks a peculiar neutrality *pH* (isoelectric point), while keeping inside the folded particle a highly hydrophobic status that helps in stabilizing the structure and the protein-protein and low electrostatic nucleic acid-protein interactions.

### 3.3. NPs Analysis: Compact Model and Virus Dynamics in the Droplets

As illustrated in previous sections, the NP behaviors of the plant virus droplet-based system were found to depend on the virus type and concentrations. In this section more insights will be reported on the NP behavior as representative of *AC* electric field-mediated mechanisms affecting the virus particle dynamics in the droplets, providing that the viruses are sensitive to the *AC* polarizing field. As for the latter requirement, this has already been assessed in a previous section. The NP simulation is accomplished by designing a suitable electrical circuit whose impedance is able to replicate the experimental ones, whereas a specific mechanism is assigned to each electrical component. As underlined, the viruses have a negative charge on their outer surface, being that the ToMV and TYMV pI are lower than the buffer pH. Moreover, the nanometer-sized virus dimensions allow the assumption that the droplet suspension is, likewise, a ‘charged’ nanoparticle-based system.

**Figure 6:**
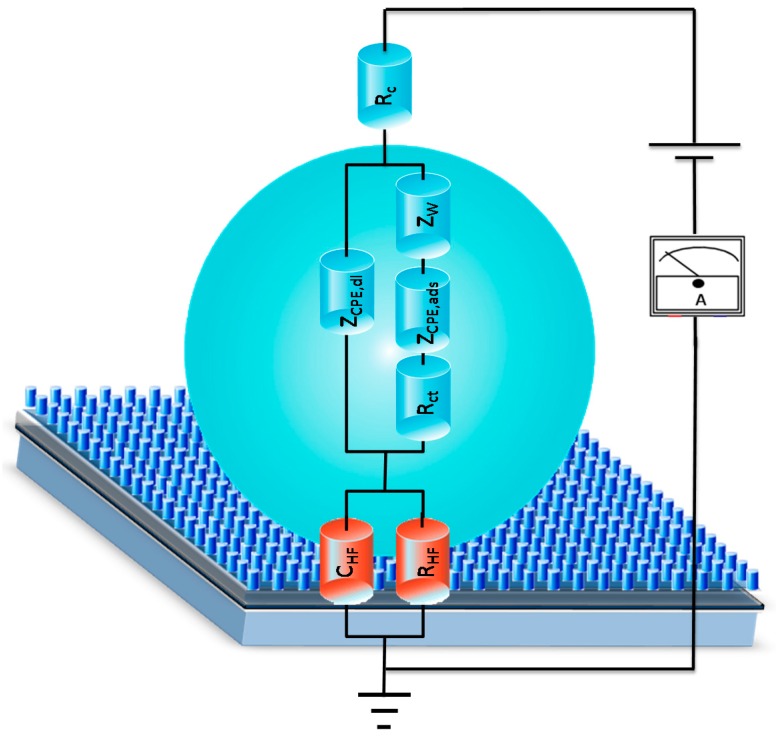
3D sketch representing the virus in a droplet system on the high hydrophobic T-pSi surface. In the figure the electrical circuit bests fitting the experimental NPs; each circuit element has been superimposed on the corresponding device structure region.

The electrical circuit representative of the High Frequency (*HF*) impedance (corresponding to the first NPs’ semicircle). of the experimentally virus-based droplets consisted of a component made up by the series of the Au-droplet contact resistance *R*_c_ with the parallel of a resistor and a capacitance labeled as *R*_HF_ and *C*_HF_ (*R*_HF_//*C*_HF_). The latter consist of the indistinguishable contribution of both the geometrical droplet suspension capacitance and resistance and that of the silicon (including the depletion width and textured region capacitance and resistance, the red part in the circuit in Figure 6) [18]. It is noteworthy that the capacitance in the presence of the virus charge, *C*_HF_, increased around two orders of magnitudes with respect to the blank solution (see Table 1). Moreover, the higher the virus concentration, the higher the magnitude of the *C*_HF_ and the lower the resistance *R*_HF_ as a result of the virus charges increasing both the droplet capacitance and the solution conductivity [20,21].

The second component was tailored to account for the in-droplet Au/droplet/T-pSi interfacial mechanisms or interaction, typically occurring at lower frequency (LF). At *V_DC_* = 0 V a classical Randle circuit adapted for surface charge adsorption was found as representative of the experimental data [26,30]. The NP in this frequency region was represented by the impedance of the droplet, *Z*_droplet_, in parallel with that of the series between the impedance of the wetted silicon textured surface, *Z*_T-pSiwet_, corresponding to the diffusion mechanism due to virus mass transfer, *Z*_W_ (*Z*_droplet_//(*Z*_T-pSiwet_ + *Z*_W_), *Z*_W_ = Warburg element). The virus-embedding droplet impedance *Z*_droplet_ contributed via an in-homogenous double layer capacitive term (constant phase element, *Z*_CPE,dl_), while the part of the droplet interacting with, or adsorbed onto, the wet textured surface was represented by the series of a non-ideal capacitive contribution (*Z*_CPE,ads_) and a resistance (*R*_ads_) (relevant best-fit parameters in Table 1).

The second component of the equivalent circuit showed increasing values of the charge surface adsorption term (*Z*_CPE,ads_) with a general decrease of the adsorption resistance, *R*_ads_ (see Table 1) with respect to blank suspension [26,30]. The double layer term *Z*_CPE,dl_ at zero bias in both virus suspensions was found to not be affected by the virus charges, (i.e., around 2.0 × 10^−8^ nF·s^(n−1)^·cm^−2^ compared to 1.5 × 10^−8^ nF·s^(n−1)^·cm^−2^ in TRIS-HCl).

As described in the previous section, the impedances of the virus suspension recorded under *DC* bias were substantially modified with respect to those at *V*_DC_ = 0 V (Figure 3) and when biasing the buffer solution (Figure 3, Figure S4). These results further highlight the impedance sensitivity to the presence of virus charges both under the *AC* and *AC* + *DC* electrical stimuli. Of note, the impedance is regulating by a Faradic behavior indicating the onset of the charge transfer reaction, offering insights on the equivalent circuit simulating the experimental NPs’ return variation in the circuital component magnitudes (see Table S2). The capacitive element *C*_HF_ was replaced by a constant phase element *Z*_CPE,HF_ while the *R*_HF_ contribution was lowered. This effect was ascribed to the effect of a *DC* bias application moving electron charges inside the droplet and returning a droplet impedance with a more marked conductive behavior.

In the second component of the equivalent circuit, the element representing the double layer, *Z*_CPE,dl_, behaves more as a resistive than a capacitive component, which can be deduced from the *n* values lower than 0.5 and also suggests that the charges layered at the Au electrode were probably unpinned.

The NP readouts under bias also gave a further route for the label-free in-droplet virus recognition. In this case, a ok electric field produced drag forces able to drift, to a different extent, the negatively-charged virus particles towards the electrode [31].

The adsorption term *Z*_CPE,abs_ increased soon after the bias application in both cases, while lowering in the ToMV droplet and remaining constant in the TYMV droplet during the time course. These results were qualitatively interpreted, due to the greater or lesser virus particle ability to penetrate into the textured surface. In this sense, considering the behavior in time of *Z*_CPE,abs_ under bias, it can be hypothesized that ToMV particles are firstly drifted by the voltage towards the textured surface, thus, increasing the adsorbed charge, and then are able to diffuse into the textured surface (leading to the decrease of the adsorbed charges at *t* = 20 min). Conversely, the TYMV particles are probably strongly agglomerating during their drift towards the textured surface (*Z*_CPE,abs_), and remain adsorbed onto the textured surfaces without diffusing (*Z*_CPE,abs_ constant value). The SEM pictures partially support the data interpretation and provide evidence of ToMV particles found inside the textured surface while no TYMV ones were observed (see SEM images in Supplementary Materials, Figure S5).

The onset and magnitude of the faradic behavior, not present in blank TRIS-HCl, hints at the presence of charge exchange current, *i*_ct_ and of a corresponding charge transfer resistance *R*_ct_, which was found related to the virus-type suspension (see Figure 3).

The ToMV-based droplet R_ct_ was found to be lower than in TYMV, indicating a higher charge exchange current (*R*_ct_ ∝ *i*_ct_^−1^, see Table S3) in the former. This is likely to be ascribed to the higher ToMV virus aspect ratio and geometrical dimension, ensuring a higher surface area with a correspondingly higher charge density contribution [31,32,33].

The virus diffusion processes indicated that even the coefficient values *Z*_W_ have magnitudes and peculiar behaviors depending on virus type.

Within this framework, the virus-based suspension was considered like a system made up of nanoparticles (the viruses) moving more or less freely in the suspending medium depending on their aspect ratio, the suspending medium viscosity, and the virus polarizability under an *AC* field.

The increase of the diffusion impedance term *Z*_W_ values soon after the bias application and during the droplet residence time suggested, again, a progressive reduction of the virus charge mobility [29,31,34]. It is noteworthy to recall that:

(6) $$Z_{W}=\frac{RT}{An^{2}F^{2}\rho_{M}\sqrt{2D}}$$

with *D* being the diffusion coefficient, R is the gas constant, T is the temperature in Kelvin (K), A is the electrode area, n is the valence of the species (*n* = 2 in this case), F is the Faraday constant, and *ρ*_M_ is the molar concentration. Findings on nanoparticle dynamics in liquid suspension showed that the diffusion coefficient relevantly differs depending on the nanoparticle aspect ratios [35]. In particular, in a suspended liquid with viscosity *η*_s_, and in a highly-diluted regime, the particles are able both to rotate freely without interacting each other due to the large distance and to diffuse due to the Brownian forces only (~*K*_B_*T*). The Brownian rotational and translation diffusion coefficients are defined as *D*_r,B_ and *D*_t,B_; for nearly spherical particles of diameter *d*, given by:

(7a) $$D_{r,B}=\frac{k_{B}T}{\left(\pi \eta_{s}d^{3}\right)}$$

(7b) $$D_{t,B}=\frac{k_{B}T}{\left(6\pi \eta_{s}R\right)}$$

while for a rod-like particle of length *L* and diameter *d*, and if the aspect ratio *L*/*d* is in the range 2 < *L*/*d* < 30, they are given by [36,37]:

(8a) $$D_{r,B}=\frac{3k_{B}T\left(ln\left(\frac{2L}{d}\right)-0.8\right)}{\left(\pi \eta_{s}L^{3}\right)}$$

(8b) $$D_{t,B}=\frac{3k_{B}T\left(ln\left(\frac{2L}{d}\right)-0.8\right)}{\left(3\pi \eta_{s}L\right)}$$

(with *k*_B_ being the Boltzmann constant, and *T* the absolute temperature). The Equations (7a,b) and (8a,b) indicate the dependence of the diffusion on the geometrical radius and aspect ratio. Considering the ToMV and TYMV geometrical shapes and dimensions, we estimate the corresponding rotational and translational diffusion coefficients. Due to the high dilution of TRIS-HCl in water (100 mM), we considered water as the suspending liquid and placed *η*_s_ = 10^−3^ Pa, i.e., the water viscosity. The *D*_t,B_ values were found to be 1.45 × 10^−11^ m^2^·s^−1^ and 0.88 × 10^−11^ m^2^·s^−1^, while the *D*_r,B_ was found to be 4.8 × 10^4^ s^−1^ and 292 s^−1^ in TYMV and ToMV, respectively. Following these results, TYMV translational and rotational diffusivity were found to be 1.6 and 160 times higher than in ToMV, respectively. Furthermore, under Brownian forces, the diffusivity does not depend on the particle concentration. 

**Figure 7:**
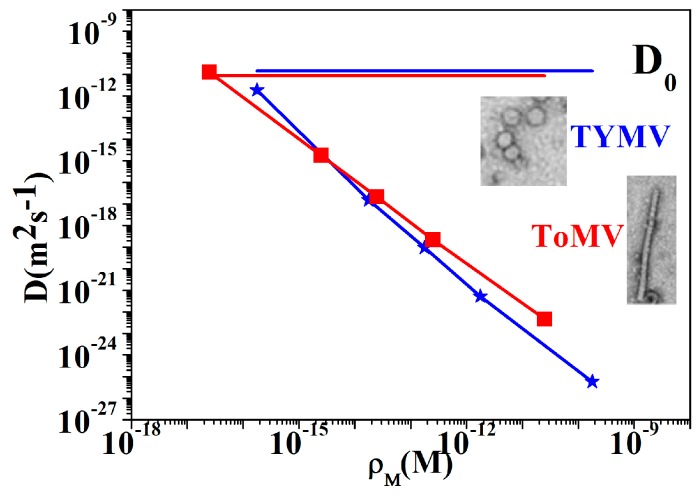
Comparison between translational diffusion coefficients (*D*) extracted from Warburg impedance (Equation (6)) at different virus molar concentrations of ToMV (red squares symbols and lines) and TYMV (blue stars symbols and lines) and the corresponding theoretically-expected ones for nanoparticles diffusion driven by Brownian forces *D*_t,B_ = *D*_0_ (from Equations (7b) and (8b)) (horizontal lines red ToMV, blue TYMV).

By representing the experimental *D* values obtained from Equation (6) (see Figure 7), it can be inferred that the at the lowest molar concentration, the diffusivity of TYMV and ToMV agree with the translational Brownian values (*D*_0_ = *D*_t,B_). Afterwards, the diffusivity progressively reduces up to ten orders of magnitude with respect to *D*_0_ with the molar concentration. This effect is commonly found in suspension embedding nanoparticles and is generally referred to as “diffusion hindering” [33], whose origin is still unclear. Although our deductions are, at this stage, not exhaustive, our results let one assume the presence of the hindering is a consequence of a drag force due to a different virus nanoparticle polarization (charging/discharging) in the buffered suspension under the applied AC field. The final effect is the limiting of the free nanoparticle Brownian motion in the buffer, in a greater or lesser extent, depending on the different virus polarization capabilities (see Figure 5).

## 4. Conclusions

The present work demonstrates a feasible and practical route for label-free and fast detection of plant viruses in aqueous medium suspension through an impedance method. The EIS technique was applied to a water-gated MIS-like device built on a hybrid droplet on a highly-hydrophobic surface, where the droplet was constituted by a suspension of virus in a TRIS-HCl solution at different virus concentrations. The virus quantification was derived by the extraction of a calibration curves in the low-frequency region of the *AC* voltage (0.1 Hz) while the virus recognition was made via the definition of a parameter specifically related to the viruses’ polarizability.

Two well-known plant viruses (ToMV and TYMV), were considered as model nanostructured systems with different substructures, shapes, and controlled surface net charge. Specifically, the potential to detect very low molar virus concentration and to extract a specific parameter allowing the label-free virus identification via a single real-time measurement was demonstrated. The method showed a peculiar sensitivity toward lowest virus concentration and selectivity to charged virus when applying a *DC* bias. We also demonstrated that the impedance behavior, and specifically the diffusivity in the suspension, were related to the virus aspect ratio.

In principle, provided that viruses are responding to an *AC* field, this method could be extended also to other droplet-shaped suspensions embedding one purified virus, since it would be an easy task to relate the parameter variations to the corresponding concentration and virus type, therefore leading to a set of calibrated curves for virus recognition. However, for a reliable applicability of the elicited approach, further validations are required as that via quantification/recognition of a much wider class of plant viruses with different shapes. Nonetheless, the presented results, although not exhaustive due to the elementary and few virus model systems examined up to now, (i.e., purified viruses in aqueous buffer solution), pave a possible route for increasing the device sensitivity and test speed by using droplet-based devices engineered for label-free plant virus detection, quantification, and recognition.

An interesting approach could also be the testing of a single droplet of sap extracted from plant tissues, which is necessary to prime the test, while reducing the cost, as it does not requires any antiserum or labelling and uses an inexpensive active electrode surface. A potential application of the EIS on our hybrid droplet-on-surface device can be envisaged even to samples with multiple virus infections or at different stages of the infection and, in the crude plant sap, assured that baseline reference measures of the expected targets have been recorded.

Considering that standard DAS-ELISA detects virus protein from 1 to10 ng/mL of sap [38], while the most sensitive RT-PCR test is able to detect femtogram amounts of virus RNA [39], our device threshold detection limit (1.0 pg/mL corresponding to 25 aM or 177 aM in ToMV and TYMV, respectively) could be considered at the border of current technical possibility with the advantage of being user-friendly and manageable by people without any specific expertise. The achievement of device portability is a challenge for the in-field tests, and remains an open issue even if, in principle, it would be possible to engineer a portable impedance apparatus that works with a similar droplet-based sensor for a rapid check at least on plant sap. The quite relevant impedance variation starting from a virus molar concentration of 25 aM also anticipates a trend towards further lowering the currently-declared detection limit.

## Figures and Tables

**Table 1:** EIS relevant best-fit parameters for T-pSi exposure to virus droplets at the virus molar concentration under test and *V*_DC_ = 0.0 V (see text and Figure 2 and Figure 6). Best fit parameters extracted from NP readouts for TRIS-HCl droplet at 0.0 V are reported for comparison.

**ToMV**					
***ρ*_M_ (M)**	***C*_HF_ (nF)**	***R*_HF_ (MΩ)**	***R*_ads_ (kΩ)**	*Z*_CPE,ads_ *	***Z*_W_^§^**
25 aM	33	0.7	10	2.5 (0.99)	104
250 aM	36	1.1	6.8	8.0 (0.94)	88
2.5 fM	48	1.0	6.4	12 (0.94)	81
25 fM	74	0.9	5.8	17 (0.93)	79
25 pM	80	1.5	13	32 (0.90)	55
TRIS-HCl	0.86 (0.82)	4.1	-	1.9 × 10^−3^ (1)	
**TYMV**					
***ρ*_M_ (M)**	***C*_HF_ (nF)**	***R*_HF_ (MΩ)**	***R*_ads_ (kΩ)**	*Z*_CPE,ads_ *	***Z*_W_^§^**
177 aM	12	2.9	14	4.9(0.94)	38
17.7 fM	33	1.6	6.8	9.1 (0.95)	135
177 fM	80	1.0	5.4	21 (0.91)	170
1.77 pM	97	1.1	6.5	25 (0.90)	228
177 pM	99	1.2	7.5	29 (0.90)	218
TRIS-HCl	0.86 (0.82)	4.1	-	1.9 × 10^−3^ (1)	

* nF·s^(n−1)^·cm^−2^; ^§^ kΩs^−0.5^.

## Supplementary Materials

The following are available online at http://www.mdpi.com/1424-8220/16/11/1946/s1.

## References

[1] Clark M.F., Adams A.N. (1977). Characteristics of the microplate method of enzyme-linked immunosorbent assay for the detection of plant viruses. J. Gen. Virol..

[2] Makkouk K.M., Hsu H.T., Kumari S.G. (1993). Detection of three plant viruses by dot-blot and tissue-blot immunoassays using chemiluminescent and chromogenic substrates. J. Phytopathol..

[3] Fenby N.S., Scott N.W., Slater A., Elliott M.C. (1995). PCR and non-isotopic labeling techniques for plant virus detection. Cell. Mol. Biol..

[4] McLaughlin M.R., Barnett O.W., Burrows P.M., Baum R.H. (1981). Improved ELISA conditions for detection of plant viruses. J. Virol. Methods.

[5] Mullis K.F., Faloona F., Scharf S., Saiki R., Horn G., Erlich H. (1986). Specific enzymatic amplification of DNA in vitro: The polymerase chain reaction. Cold Spring Harb. Symp. Quant. Biol..

[6] Henson J.M., French R. (1993). The polymerase chain reaction and plant disease diagnosis. Annu. Rev. Phytopathol..

[7] Eun A.J.-C., Huang L., Chew F.T., Li S.F.-Y., Wong S.M. (2002). Detection of two orchid viruses using quartz crystal microbalance (QCM) immunosensors. J. Virol. Methods.

[8] Boltovets P.M., Snopok B.A., Boyko V.R., Shevchenko T.P., Dyachenko N.S., Shirshov Y.M. (2004). Detection of plant viruses using a surface plasmon resonance via complexing with specific antibodies. J. Virol. Methods.

[9] Mendes R.K., Carvalhal R.F., Stach-Machado D.R., Kubota L.T. (2009). Surface plasmon resonance immunosensor for early diagnosis of Asian rust on soybean leaves. Biosens. Bioelectron..

[10] Fumagalli L., Esteban-Ferrer D., Cuervo A., Carrascosa J.L., Gomila G. (2012). Label-free identification of single dielectric nanoparticles and viruses with ultraweak polarization forces. Nat. Mater..

[11] Ahmad M.A., Mustafa F., Ali L.M., Rizvi T.A. (2014). Virus detection and quantification using electrical parameters. Sci. Rep..

[12] Al Ahmad M., Mustafa F., Ali L.M., Karakkat J.V., Rizvi T.A. (2015). Label-free capacitance-based identification of viruses. Sci. Rep..

[13] Birnbaumer G.M., Lieberzeit P.A., Richter L., Schirhagl R., Milnera M., Dickert F.L., Bailey A., Ertl P. (2009). Detection of viruses with molecularly imprinted polymers integrated on a microfluidic biochip using contact-less dielectric microsensors. Lab Chip.

[14] Jarocka U., Radecka H., Malinowski T., Michalczuk L., Radecki J. (2013). Detection of Prunus Necrotic Ringspot Virus in Plant Extracts with Impedimetric Immunosensor Based on Glassy Carbon Electrode. Electroanalysis.

[15] Cahill B.P., Wiedemeier S., Gastrock G. (2013). Measurement of droplet impedance in segmented flow. *Tagungsband*, Proceedings of Dresdner Sensor-Symposium 2013.

[16] Simon M.G., Lin R., Lopez-Prieto J., Lee A.P., Landers J.P., Bienvenue J., Herr A. (2011). Label-free detection of dna amplification in droplets using electrical impedance. Proceedings of the 15th International Conference on Miniaturized Systems for Chemistry and Life Sciences 2011 (MicroTAS 2011).

[17] Wang X., Ma S., Su Y., Zhang Y., Bi H., Zhang L., Han X. (2015). High Impedance Droplet–Solid Interface Lipid Bilayer Membranes. Anal. Chem..

[18] Dak P., Ebrahimi A., Alam M.A. (2014). Non-faradaic impedance characterization of an evaporating droplet for microfluidic and biosensing applications. Lab Chip.

[19] Ebrahimi A., Dak P., Salm E., Dash S., Garimella S.V., Bashir R., Alam M.A. (2013). Nanotextured superhydrophobic electrodes enable detection of attomolar-scale DNA concentration within a droplet by non-faradaic impedance spectroscopy. Lab Chip.

[20] Turnip Yellow Mosaic Virus. http://www.dpvweb.net/dpv/showdpv.php?dpvno=230.

[21] Tomato Mosaic Virus. http://www.dpvweb.net/dpv/showdpv.php?dpvno=156.

[22] Foster G.D., Taylor S.C. (1998). Plant Virology Protocols: From Virus Isolation to Transgenic Resistance.

[23] Brakke M.K., Maramorosch K., Koprowski H. (1967). Density-Gradient Centrifugation. Methods in Virology 2.

[24] Di Mundo R., Ambrico M., Ambrico P.F., D’Agostino R., Italiano F., Palumbo F. (2011). Single-Step Plasma Process Producing Anti-Reflective and Photovoltaic Behavior on Crystalline Silicon. Plasma Process. Polym..

[25] Ambrico M., Ambrico P.F., Cardone A., Cicco S.R., Palumbo F., Ligonzo T., Di Mundo R., Petta V., Augelli V., Favia P. (2014). Melanin-like polymer layered on a nanotextured silicon surface for a hybrid biomimetic interface. J. Mater. Chem. C.

[26] Bondarenko A.S., Ragoisha G.A., Osipovich N.P., Streltsov E.A. (2005). Potentiodynamic electrochemical impedance spectroscopy of lead upd on polycrystalline gold and on selenium atomic underlayer. Electrochem. Commun..

[27] Bondarenko A.S., Ragoisha G.A. EIS Spectrum Analyser. http://www.abc.chemistry.b-su.by/vi/analyser/.

[28] Barsoukov E., Macdonald J.R. (2005). Impedance Spectroscopy: Theory, Experiment, and Applications.

[29] Song J., Bazant M.Z. (2012). Effects of Nanoparticle Geometry and Size Distribution on Diffusion Impedance of Battery Electrodes. J. Electrochem. Soc..

[30] Maxakato N.W., Ozoemena K.I., Arendse C.J. (2010). Dynamics of Electrocatalytic Oxidation of Ethylene Glycol, Methanol and Formic Acid at MWCNT Platform Electrochemically Modified with Pt/Ru Nanoparticles. Electroanalysis.

[31] Yun H.J., Lee H., Joo J.B., Kim W., Yi J. (2009). Influence of Aspect Ratio of TiO_2_ Nanorods on the Photocatalytic Decomposition of Formic Acid. J. Phys. Chem. C.

[32] Bonanni A., Pumera M., Miyahara Y. (2011). Influence of gold nanoparticle size (2–50 nm) upon its electrochemical behavior: An electrochemical impedance spectroscopic and voltammetric study. Phys. Chem. Chem. Phys..

[33] Woehl T.J., Prozorov T. (2015). The Mechanisms for nanoparticle surface diffusion and chain self-assembly determined from real-time nanoscale kinetics in liquid. J. Phys. Chem. C.

[34] Moore W.J. (1978). Physical Chemistry.

[35] Cassagnau P. (2013). Linear viscoelasticity and dynamics of suspensions and molten polymers filled with nanoparticles of different aspect ratios. Polymer.

[36] Sergio R.A. (1987). Forward Depolarized Light Scattering from Wormlike Chains. Macromolecules.

[37] Senyuk B., Glugla D., Smalyukh I.I. (2013). Rotational and translational diffusion of anisotropic gold nanoparticles in liquid crystals controlled by varying surface anchoring. Phys. Rev. E Stat. Nonlinear Soft Matter Phys..

[38] Bergmeyer H.U. (1986). Plant Viruses. Methods of Enzymatic Analysis, Volume XI. Antigens and Antibodies 2.

[39] Candresse T., Hammond R.W., Hadidi A., Hadidi A., Khetarpal R.K., Koganezawa H. (1998). Detection and identi-fication of plant viruses and viroids using polymerase chain reaction (PCR). Plant Virus Disease Control.

