# Supplementary material for: Highly Sensitive and Practical Detection of Plant Viruses via Electrical Impedance of Droplets on Textured Silicon-Based Devices
Source: Sensors (Basel). 2016 Nov 18;16(11):1946. doi: 10.3390/s16111946 (PMC5134605; doi:10.3390/s16111946)
Supplement: Supplementary file 1 [file sensors-16-01946-s001.pdf]

Supplementary Materials: Highly Sensitive and Practical Detection of Plant Viruses via Electrical Impedance of Droplets on Textured Silicon-Based Devices

Marianna Ambrico, Paolo Francesco Ambrico, Angelantonio Minafra, Angelo De Stradis, Danilo Vona, Stefania R. Cicco, Fabio Palumbo, Pietro Favia and Teresa Ligonzo

Table S1. Chemical-physical properties of TYMV and ToMV: Average yields of purified viruses (in mg/ml of suspension) was accordingly converted into molar concentration to calculate the sensitivity threshold limit.

Virus	Average Purified Amount (mg/mL)	Nucleic Acid Content (%)	$A_{260/280}$	Extinction Coefficient *	MW of a Virion †	Coat Protein Subunit MW	CP Subunits per Particle	Molar Concentration (mol/mL) ‡
TYMV	12	35	1.78	7.69§	5.6×10^6 Da	20.13 kDa	180	1.77×10^{-19} mol/mL
ToMV	15	5	1.25	3.1	3.96×10^7 Da	18.0 kDa	2130	2.52×10^{-20} mol/mL

* Absorbance of a purified virus suspension at 1 mg/mL at 260 nm UV light. † Including genomic RNA molecule; ‡ For 1 pg/mL virus; § Average of the bottom and top component [1].

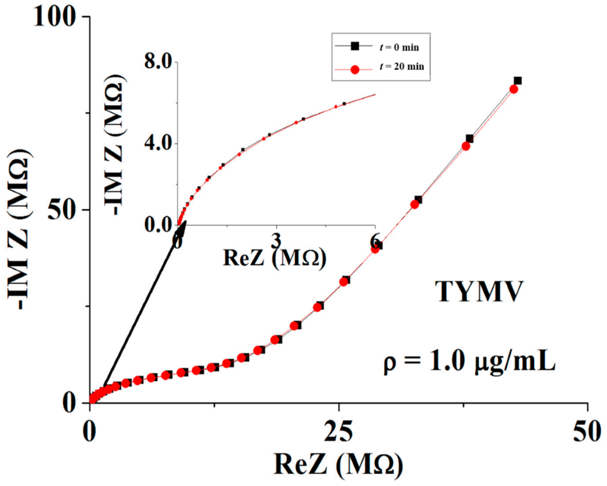

Figure S1. Evolution of NPs at V = 0 V for virus-in TRIS-HCl droplet collected at t = 0 min and subsequently read outs up to a residence time t = 20 min showing the same magnitude of the impedance values thus excluding possible relevant droplet evaporation. Similar results were obtained also in blank Tris-HCl. The inset is an enlarged view of the HF region.

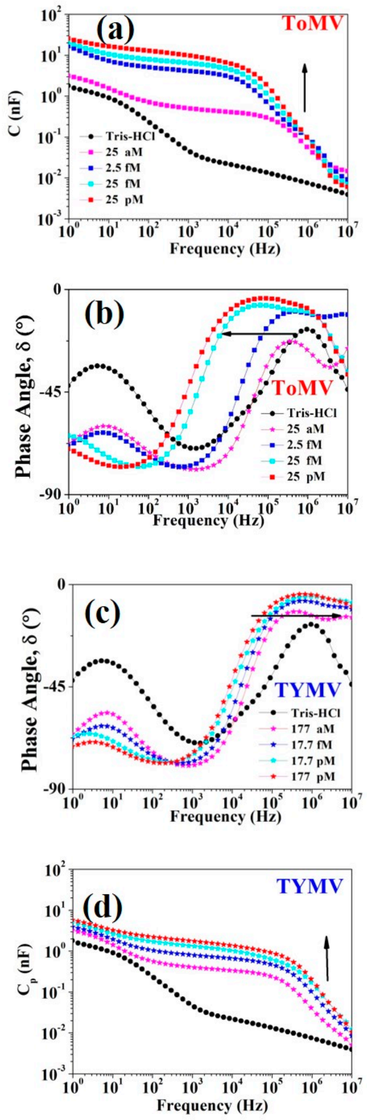

Figure S2. Capacitance vs. frequency at different molar concentrations and phase angles for ToMV (**a**,**b**) and TYMV (**c**,**d**) virus based suspension.

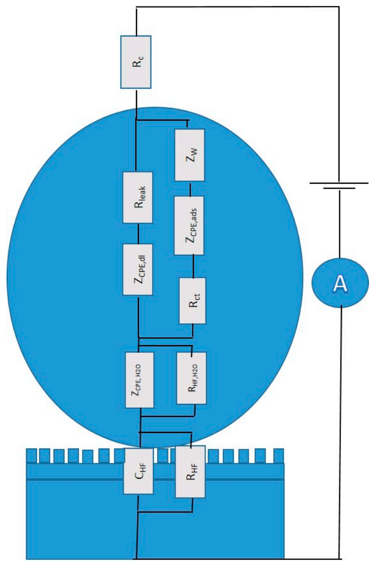

Figure S3. Sketch of modified Randles circuit for NPs representing blank TRIS-HCl droplet on the T-Si surface system.

Table S2. EIS best-fit parameters variations for a T-Si exposure to droplets with different virus concentration and V_{DC} = 0.0 V (see text) for ToMV/TRIS-HCl (up) and TYMV/TRIS-HCl (down). The best fit parameters extracted from NP of T-Si surface exposure to TRIS-HCl droplet are reported for comparison.

ρ (ToMV) Fitting Parameters	1.0 pg/mL	0.1 ng/mL	1.0 ng/mL	10 ng/mL	1.0 μg/mL	Tris-HCl
C_{HF}	2.5 × 10^{-8}	3.65 × 10^{-8}	4.85 × 10^{-8}	7.4 × 10^{-8}	8.1 × 10^{-7}	8.6 × 10^{10}, 0.8 *
R_c	1097	6000	7090	6286	1097	
R_{HF}	7.0 × 10^5	1.13 × 10^{-6}	1.01 × 10^6	9.5 × 10^5	1.5 × 10^6	4.1 × 10^6
R_{ads}/R_{ct}	11,036	6866	6456	5886	13,067	-
$Z_{CPE,dl}$	1.76 × 10^{-8}	2.023 × 10^{-8}	2.05 × 10^{-8}	1.89 × 10^{-8}	7.5 × 10^{-9}	1.5 × 10^{-8}
n_1	0.64	0.62	0.59	0.58	0.5	0.63
$Z_{CPE,ads}$	2.5 × 10^{-9}	8.1 × 10^{-9}	1.22 × 10^{-8}	1.64 × 10^{-8}	3.1 × 10^{-8}	1.9 × 10^{-12}
n_2	0.99	0.94	0.94	0.93	0.9	1
Z_W	0.0	88.654	81,075	77,831	55,477	0.0

* $Z_{CPE,HF(1MHz)}$, n.

ρ (TYMV) Fitting Parameters	1.0 pg/mL	0.1 ng/mL	1.0 ng/mL	10 ng/mL	1.0 μg/mL	Tris-HCl
C_{HF}	1.2 × 10^{-8}	3.3 × 10^{-8}	1.2 × 10^{-7}	1.15 × 10^{-7}	1.2 × 10^{-8}	8.6 × 10^{-10} †, 0.8 †
R_c	7740	7009	5714	4660	3382	
R_{HF}	3.2 × 10^6	1.61 × 10^6	6.8 × 10^5	6.7 × 10^5	8.2 × 10^5	4.1 × 10^6
R_{ads}/R_{ct}	14,285	6868	5450	6502	7549	-
$Z_{CPE,dl}$	1.6 × 10^{-8}	1.8 × 10^{-8}	2.1 × 10^{-8}	2.0 × 10^{-8}	1.47 × 10^{-8}	1.5 × 10^{-8}
n_1	0.58	0.54	0.54	0.52	0.52	0.63
$Z_{CPE,ads}$	5.1 × 10^{-9}	9.1 × 10^{-9}	1.5 × 10^{-8}	2.6 × 10^{-8}	2.6 × 10^{-8}	1.9 × 10^{-12}
n_2	0.94	0.95	0.95	0.94	0.9	1
Z_W	38.170	1.35 × 10^5	2.81 × 10^5	2.6 × 10^5	86.968	0.0

† $Z_{CPE,HF(1MHz),n}$, $Z_{CPE} = \frac{1}{Q(j\omega)^n}$ (nF·$s^{(n-1)}$/cm^2); $\frac{A_W}{(j\omega)^{0.5}}$ (Ω·$s^{-0.5}$).

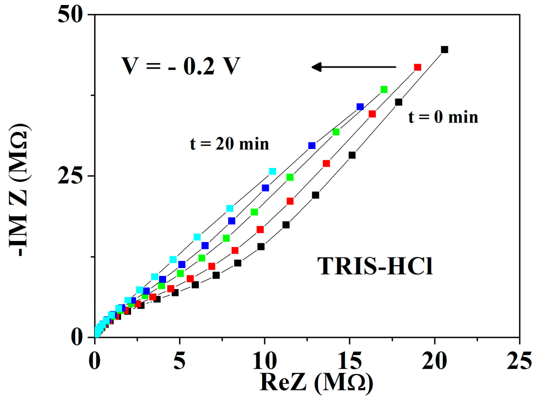

Figure S4. Evolution of NPs at V = −0.2 V for blank TRIS-HCl droplet collected consecutively for residence time t_r = 20 min showing a shift in time opposite to that observed in virus in TRIS-HCl droplet under similar conditions.

SEM Images of textured surfaces: SEM images (Figure S5) have been acquired by means of a Supra 40 SEM-FEG (Zeiss, Oberkochen, Germany) with an extraction voltage of 5 KV, at 0° of tilting angle, both on the top of the samples and from cross sections obtained by smoothly breaking the silicon sample.

(**a**)

(**b**)

(**c**)

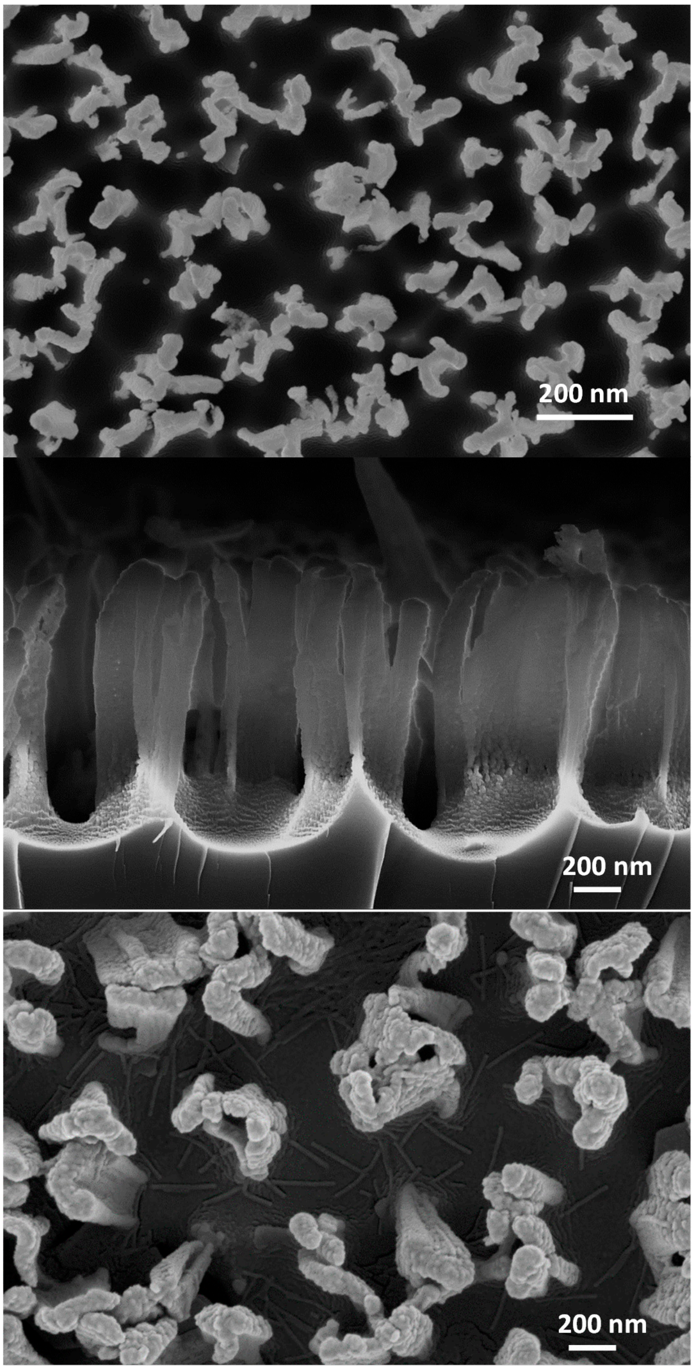

Figure S5. (**a**) Top image of an as prepared T-pSi surface and (**b**) corresponding cross section. It can be seen that the surface is composed of homogeneously distributed islands with lateral size and distance around 0.3 and 0.5 µm. However looking at the cross section it is possible to observe that such islands are as tall as 1.1 µm, and composed of thinner units (**c**) T-pSi is exposed to a drop of ToMV (0.1 mg/mL), and dried in a vacuum chamber; the virus can be layered on the bottom of the sample between the islands. (Figure S5c) In the case of **v**irus incubated samples, the SEM analysis was carried out onto 20 nm gold metalized surfaces.

References

1. Descirptions of Plant Viruses. Available online: http://www.dpvweb.net (acccessed on 14 November 2016).
